# Ablation of cervical inlet patch for the treatment of globus sensation: A case report

**DOI:** 10.1002/ccr3.8074

**Published:** 2023-11-13

**Authors:** Arnav Shah, Timothy Fan, Aaron Jaworek

**Affiliations:** ^1^ Lewis Katz School of Medicine at Temple University Philadelphia Pennsylvania USA; ^2^ Department of Otolaryngology—Head and Neck Surgery St. Luke's University Health Network Bethlehem Pennsylvania USA

**Keywords:** bronchoesophagology, cervical inlet patch, dysphagia, esophageal inlet patch, esophagoscopy, globus, heterotopic gastric mucosa, laryngopharyngeal reflux, laser ablation

## Abstract

We present a case of a medically resistant cervical inlet patch causing persistent globus and symptoms of laryngo‐pharyngeal reflux, successfully treated with CO2 laser ablation.

## Graphical abstract

Cervical inlet patch immediately following endoscopic CO2 laser ablation.

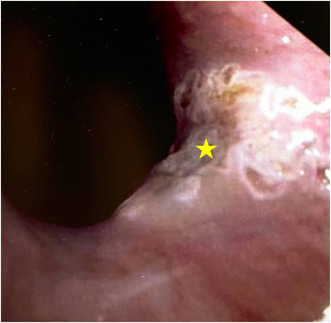

## 1 INTRODUCTION

Cervical inlet patch (CIP) represents an island of heterotopic gastric mucosa typically located in the proximal esophagus, with an estimated prevalence rate of 0.1%–18.0%.^1^ Due to its proximity to the hypopharynx and larynx, CIP may present with symptoms similar to laryngopharyngeal reflux (LPR), while others can be asymptomatic.^1^ The histology of CIP also lends itself to acid reflux related findings, including esophagitis, esophageal stricture, esophageal perforation, and rarely, progression to Barrett's esophagus and adenocarcinoma.^1^, ^2^, ^3^, ^4^ Many symptoms associated with CIP overlap with LPR, including globus sensation (1.6%–78.6%), dysphagia (15.2%–39.4%), and chronic cough (29.2%).^2^, ^5^ We report on a case of refractory globus sensation determined to be due to a CIP, as well as a new treatment technique using carbon dioxide (CO2) laser ablation with a Weerda laryngoscope.

## 2 CASE REPORT

A 54‐year‐old woman presented to the laryngology office with a sensation of lump in the throat (globus sensation), sore throat, as well as intermittent hoarseness and pill dysphagia. She had been taking lansoprazole 30 mg every morning for many months without perceived benefits. Allergy evaluation for regional aeroallergens and food allergens was negative. A gluten free diet provided some benefits and was continued by the patient. Voice therapy by a subspecialized speech‐language pathologist had been initiated by the referring otolaryngologist. Her Reflux Symptom Index was 5 (normal).

**FIGURE 1:**
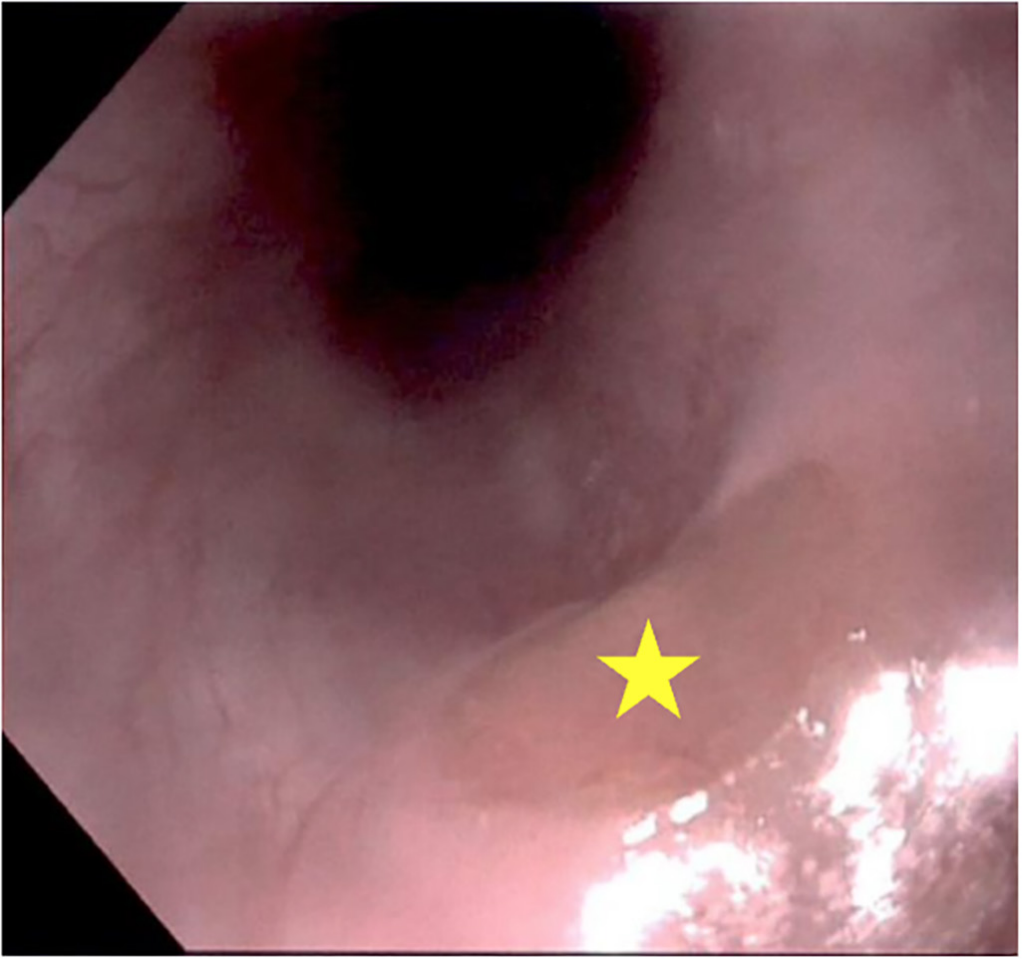
On esophagogastroduodenoscopy, a cervical inlet patch (star) is noted in the posterior upper esophagus with a smooth, raised, well‐circumscribed area of gastric mucosa, distinct from the surrounding esophageal mucosa.

A comprehensive head and neck physical exam was unremarkable. Flexible fiberoptic laryngoscopy with stroboscopy revealed evidence of mild reflux laryngitis (Reflux Finding Score = 9), moderate muscle tension dysphonia (MTD), and mild right vocal fold hypomobility. A 24‐h pharyngeal pH probe off reflux medications was obtained which demonstrated 63 reflux events mostly overnight/supine, 16 with a pH <5. Globus sensation correlated with reflux 3 of 3 times including one episode with pH 5–5.5, and throat clearing correlated 2 of 3 times. Famotidine 40 mg at bedtime with alginate therapy and reflux diet/lifestyle modifications were prescribed. This improved her symptoms further; however, they had not resolved. A referral to gastroenterology was made for evaluation with esophagogastroduodenoscopy (EGD) and possible reflux surgical discussion. During her EGD, a CIP in the upper esophagus at the level of the cricopharyngeus muscle was identified (Figure 1). The remainder of the EGD was essentially normal including biopsies of the proximal, mid, and distal esophagus (<3 eosinophils per high power field), stomach, and duodenum.

**FIGURE 2:**
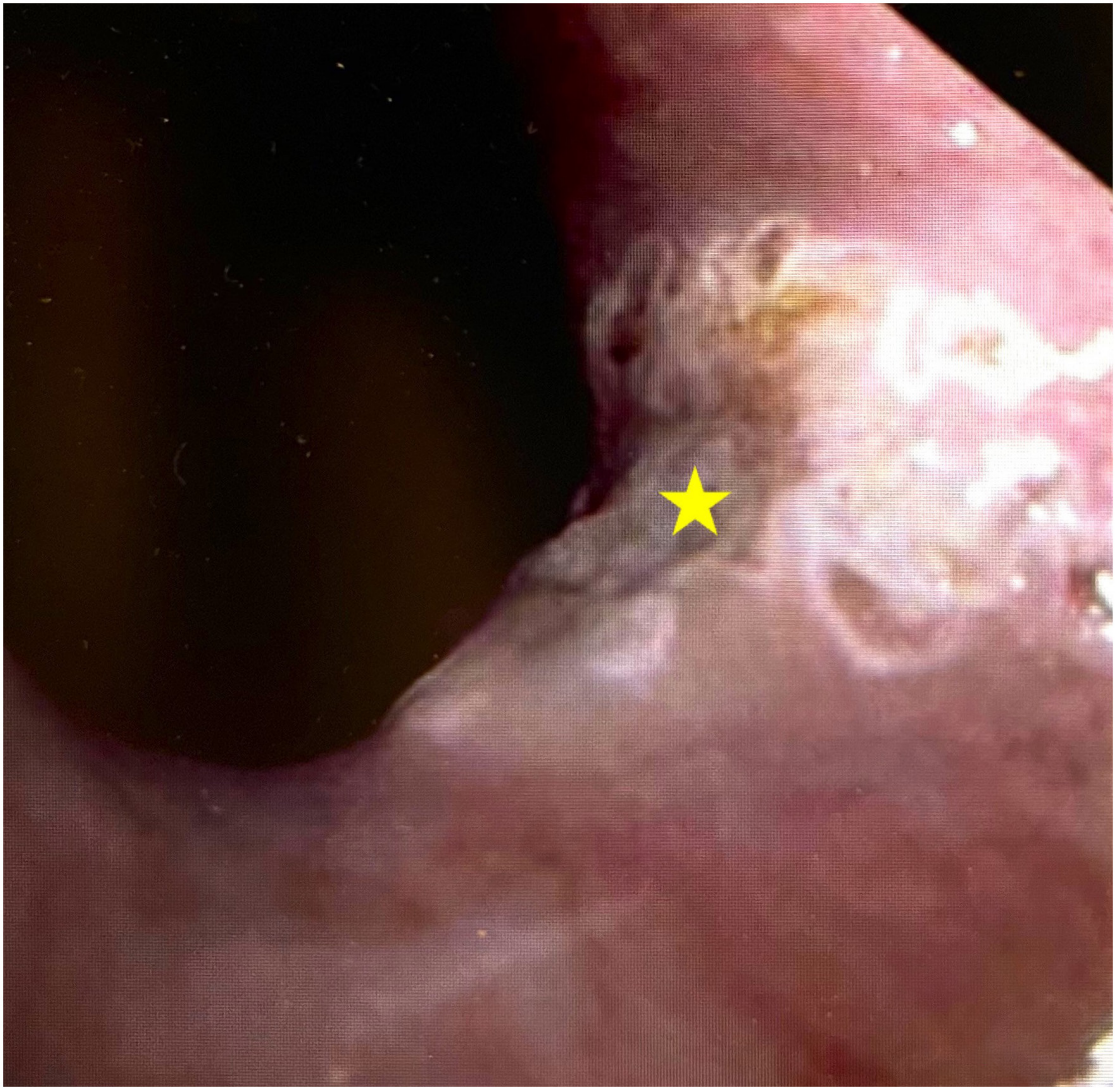
On endoscopic examination, the cervical inlet patch (star) is visualized with postoperative changes from the carbon dioxide laser ablation.

The patient was offered ablation of the CIP to treat her persistent globus sensation. She underwent micro direct laryngoscopy (MDL) using a Weerda laryngoscope and CO2 laser ablation of the CIP via laser fiber with rigid handpiece (Figure 2). The patient reported complete resolution of her globus sensation 3 days post‐procedure. Following the procedure, the patient additionally reported no dysphagia, no heart burn sensations, no sore throat (except for mild throat discomfort in the first 5 days post‐operation, presumably due to the procedure), and minimized intermittent hoarseness (only present at the end of the day following excessive voice usage). Post procedure, the patient did not receive any additional proton pump inhibitor therapy. In the immediate three‐day postoperative period, she remained on famotidine therapy, which was weaned over the next 12 months. Follow up over a > 12‐months period confirmed absence of her globus sensation and other laryngeal complaints. She had weaned off antisecretory reflux medications, but continued gluten reduced diet and avoidance of reflux trigger foods during this time frame.

Institutional Review Board (IRB) exemption was obtained for this case report from the St. Luke's University Health Network IRB.

## 3 DISCUSSION

For patients with globus refractory to medical therapy, it is important to consider a CIP as the causative pathology. An upper gastrointestinal endoscopy is the principle diagnostic tool.^2^ To date, no standard treatment recommendations have been defined for CIP. For asymptomatic or mild cases, active monitoring and/or treatment with acid suppression therapy may be considered. More aggressive treatment options have been described for symptomatic CIP, including endoscopic ablation with argon plasma coagulation (APC), radiofrequency ablation (RFA), and endoscopic excision. In one prospective randomized controlled trial, subjects reported resolution of globus sensation with APC without any adverse stricture formation.^6^ Similarly, multiple smaller prospective trials utilizing RFA have reported successful resolution of globus sensation, dysphagia, and cough.^2^, ^3^, ^7^, ^8^ Lastly, for cases of high grade dysplasia or adenocarcinoma originating from CIP, endoscopic resection remains a therapeutic option for early stage cancers.^2^

This case describes MDL with CO2 laser ablation as a viable treatment option for CIP. In the gastrointestinal literature, CO2 lasers are documented as a safe and efficacious method for endoscopic submucosal resection for early gastrointestinal neoplasms. Compared to APC and RFA that carry a risk of stricture formation, bleeding, and perforation, CO2 laser ablation has a lower risk of perforation and thermal damage.^2^, ^4^, ^9^, ^10^ As the CO2 laser energy is absorbed by water, it allows for ablation of the heterotrophic mucosa concurrently with hemostasis. The CO2 laser ablation completely resolved the patient's globus symptom without any complications. There was no recurrence of her symptoms after >12 months of monitoring, demonstrating its safety and efficacy.

## 4 CONCLUSION

Symptoms of CIP vary widely and may mimic symptoms associated with LPR. While CIP is a relatively benign condition, delayed diagnosis may induce significant disruption to quality of life. Through this case presentation, the authors hope to contribute to contemporary understanding of this unique entity by improving awareness of CIP as a cause of globus sensation and other LPR symptoms, along with introducing an alternative treatment approach for the otolaryngologist.

## AUTHOR CONTRIBUTIONS

**Arnav Shah:** Formal analysis; project administration; writing – original draft; writing – review and editing. **Timothy Fan:** Conceptualization; data curation; formal analysis; supervision; writing – original draft; writing – review and editing. **Aaron Jaworek:** Conceptualization; data curation; investigation; methodology; supervision; validation; visualization; writing – review and editing.

## FUNDING INFORMATION

None.

## CONFLICT OF INTEREST STATEMENT

None.

## CONSENT

Written informed consent was obtained from the patient to publish this report in accordance with the journal's patient consent policy.

## Data Availability

The data that support the findings of this study are available from the corresponding author, AJ, upon reasonable request.
